# DEVELOPMENT OF AN EVIDENCE-BASED FRAMEWORK FOR INNOVATION READINESS OF LONG-TERM CARE ORGANIZATIONS

**DOI:** 10.1093/geroni/igad104.3275

**Published:** 2023-12-21

**Authors:** Monique van den Hoed, Ramona Backhaus, Audrey Beaulen, Jan Hamers, Ramon Daniels

**Affiliations:** Maastricht University, Maastricht, Limburg, Netherlands; Maastricht University, Maastricht, Limburg, Netherlands; Maastricht University, Maastricht, Limburg, Netherlands; Maastricht University, Maastricht, Limburg, Netherlands; Maastricht University, Maastricht, Limburg, Netherlands

## Abstract

Increasing innovation readiness of long-term care organizations is vital to ensure future provision and affordability of care delivery. Against this background, there is a need to obtain more insight into the factors that may lead to innovation readiness of long-term care organizations. Therefore, we conducted a scoping review and an interview study. The scoping review was based on the framework from Arksey and O’Malley and included 44 studies. The interview study was conducted with 16 stakeholders having a professional role in long-term care for older adults in the Netherlands: academics, (top)management, innovation managers and consultants. Based on the results of both studies, we propose a framework of factors contributing to innovation readiness. The framework consists of five main factors enabling innovation readiness of long-term care organizations for older adults: 1) strategic course for innovation 2) innovation journey 3) leadership for innovation 4) learning for innovation and 5) innovative organizational culture. The collective findings support the notion that for innovation readiness the interplay of main factors is vital and benefits from an approach at the individual, team, organizational and inter-organizational levels. Furthermore, our findings indicate that some factors might be more conditional and other factors might play a more supportive role. Research into innovation readiness of health care organizations is a rather new field. Further research directed toward a framework for innovation readiness might deliver a structured approach for managers of long-term care organizations to embed and assess innovation readiness.

